# Infection of wild-caught wood mice (*Apodemus sylvaticus*) and yellow-necked mice (*A. flavicollis*) with tick-borne encephalitis virus

**DOI:** 10.1038/s41598-023-47697-2

**Published:** 2023-12-07

**Authors:** Julian W. Bakker, Emily L. Pascoe, Sandra van de Water, Lucien van Keulen, Ankje de Vries, Lianne C. Woudstra, Helen J. Esser, Gorben P. Pijlman, Willem F. de Boer, Hein Sprong, Jeroen Kortekaas, Paul J. Wichgers Schreur, Constantianus J. M. Koenraadt

**Affiliations:** 1 Laboratory of Entomology, Wageningen University & Research, Wageningen, The Netherlands; 2 Present Address: Conservation Genomics Research Unit, Research and Innovation Centre, Fondazione Edmund Mach, Trento, Italy; 3 Department of Virology and Molecular Biology, Wageningen Bioveterinary Research, Lelystad, The Netherlands; 4 Department of Bacteriology, Host-Pathogen Interaction and Diagnostics Development, Wageningen Bioveterinary Research, Lelystad, The Netherlands; 5 National Institute of Public Health and the Environment (RIVM), Utrecht, The Netherlands; 6 Wildlife Ecology and Conservation Group, Wageningen University & Research, Wageningen, The Netherlands; 7 Laboratory of Virology, Wageningen University & Research, Wageningen, The Netherlands; 8 Present Address: Boehringer Ingelheim Animal Health, Saint Priest, France

**Keywords:** Ecology, Pathogens, Virology, Entomology

## Abstract

The distribution of tick-borne encephalitis virus (TBEV) is expanding to Western European countries, including the Netherlands, but the contribution of different rodent species to the transmission of TBEV is poorly understood. We investigated whether two species of wild rodents native to the Netherlands, the wood mouse *Apodemus sylvaticus* and the yellow-necked mouse *Apodemus flavicollis*, differ in their relative susceptibility to experimental infection with TBEV. Wild-caught individuals were inoculated subcutaneously with the classical European subtype of TBEV (Neudoerfl) or with TBEV-NL, a genetically divergent TBEV strain from the Netherlands. Mice were euthanised and necropsied between 3 and 21 days post-inoculation. None of the mice showed clinical signs or died during the experimental period. Nevertheless, TBEV RNA was detected up to 21 days in the blood of both mouse species and TBEV was also isolated from the brain of some mice. Moreover, no differences in infection rates between virus strains and mouse species were found in blood, spleen, or liver samples. Our results suggest that the wood mouse and the yellow-necked mouse may equally contribute to the transmission cycle of TBEV in the Netherlands. Future experimental infection studies that include feeding ticks will help elucidate the relative importance of viraemic transmission in the epidemiology of TBEV.

## Introduction

Tick-borne encephalitis virus (TBEV) is endemic in large parts of Europe and Asia, where it causes between 4000 and 9000 human cases of tick-borne encephalitis (TBE) each year^1^. In Europe, the virus is predominantly transmitted between *Ixodes ricinus* ticks and small mammals, particularly rodents. Ticks can acquire the virus while feeding on a viraemic host or via co-feeding (also called non-viraemic transmission) with an infected tick on a naïve or immune host^2,3^. The non-viraemic transmission route is considered the most important transmission pathway and principally occurs between infected nymphs and uninfected larvae that feed simultaneously on a rodent host^3,4^.

Rodents, such as the wood mouse *Apodemus sylvaticus*, the yellow-necked mouse *Apodemus flavicollis* and the bank vole *Myodes glareolus,* are the primary hosts of *I. ricinus* larvae and, to a lesser extent, nymphs. The relative contribution to the eco-epidemiology of TBEV likely differs between rodent species. For example, *Apodemus* mice have higher tick burdens^5^, higher tick-feeding success, and more efficiently support TBEV transmission via co-feeding compared to bank voles^2^. Whether *Apodemus* mice also develop higher or prolonged viraemia than bank voles remain to be determined. Likewise, it remains unclear if differences in infection rates and viraemia also exist among species within the genus *Apodemus*. These questions are important to address, because given that the community composition of rodents differs across the geographic distribution of TBEV^6–8^, any difference in reservoir competence among rodent species is likely to affect TBEV transmission dynamics.

In recent years, TBEV has been detected in new regions in Europe, including several Western-European countries such as the Netherlands, the United Kingdom and Belgium^9–11^. In the Netherlands, TBEV was first detected in ticks in 2015, followed by the first human cases in 2016^10,12^. After that, multiple TBEV strains have been detected in the Netherlands^13^. Some of these strains are closely related to the classical European subtype that is found in central parts of the Netherlands whereas another strain (TBEV-NL) forms a subclade of the previously known Western-European strains and has only been detected in National Park Sallandse Heuvelrug, The Netherlands, and in the United Kingdom^14^.

*Apodemus flavicollis* is widespread in Central Europe and is considered one of the most important hosts for TBEV transmission^2^. In the Netherlands, however, this species is relatively rare and its distribution is confined to the eastern parts of the Netherlands, where TBEV was first detected^15,16^. In contrast, *A. sylvaticus* is widespread in the Netherlands^15,17^. The geographic overlap between *A. flavicollis* and the first cases of TBEV raised the question whether *A. sylvaticus* and *A. flavicollis* may differentially contribute to TBEV transmission. Moreover, we hypothesized that the restricted distribution of TBEV-NL compared to classical European strains such as TBEV-Neudoerfl may be the result of reduced susceptibility of wild rodents to TBEV-NL.

Compared to co-feeding transmission, direct transmission of TBEV from viraemic hosts to ticks was previously suggested to play only a minor role in the TBEV transmission cycle, as studies showed that viraemia in rodents only lasts between 2 and 4 days^18,19^. However, recent studies showed that TBEV RNA is present in the blood of experimentally infected bank voles (*Myodes glareolus*) and common voles (*Microtus arvalis*) for over 30 days and that infectious virus can be isolated from different organs after more than 90 days^7,20^. Furthermore, the virus has been isolated from leukocytes of bank voles 14 days after inoculation^21^. This suggests that viraemic transmission of TBEV by rodents may be more important than basic reproduction models currently estimate^22^. To what degree the viraemic period, and hence the potential contribution to TBEV transmission, differs between rodent species is also largely unknown.

Here, we tested the susceptibility of two rodent hosts, *A. sylvaticus* and *A. flavicollis*, for two different TBEV strains. Animals were live trapped and inoculated with TBEV-NL or TBEV-Neudoerfl after which both systemic and local infection was followed in time for up to three weeks.

## Results

### Clinical signs and weight loss in mice after TBEV inoculation

**Figure 1:**
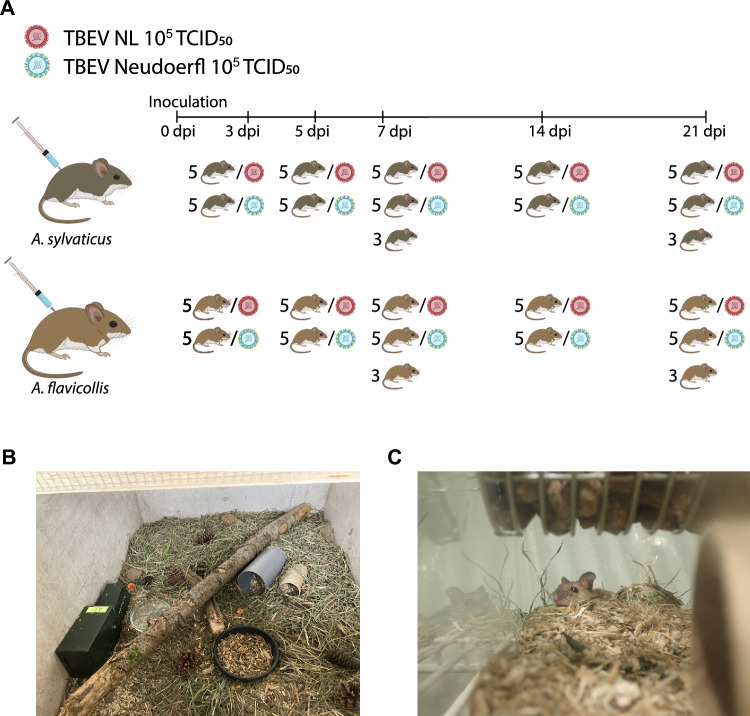
(**A**) Schematic representation of the study design. Mice were inoculated with 10^5^ TCID_50_ at day 0. Five animals per species (*A. flavicollis* and *A. sylvaticus*) per virus (TBEV-NL or TBEV-Neudoerfl) were necropsied per timepoint (3, 5, 7, 14-and 21 days post infection). Three negative control mice were necropsied at 7 and 21 dpi. (**B**) Mesocosm cage in which the animals were housed before transport to the BSL-3 facility. (**C**) An *A. flavicollis* mouse in a Makrolon IIL cage in the BSL-3 facility.

A total of 112 (51 male and 49 female) wild-caught *A. flavicollis* (n = 56) and *A. sylvaticus* (n = 56) mice were used for the TBEV infection experiments; 100 animals were subcutaneously inoculated with 10^5^ TCID_50_ of either TBEV-Neudoerfl (n = 50) or TBEV-NL (n = 50) on day 0, while 12 animals were mock infected and served as controls. All mice tested seronegative for TBEV IgG at the start of the experiment. Mice were necropsied on 3, 5, 7, 14, or 21 days post inoculation (dpi, Fig. 1A). For each timepoint, five animals were necropsied per species and per virus strain. Three *A. flavicollis* and three *A. sylvaticus* mice were necropsied at 7 and 21 dpi as negative control animals.

**Figure 2:**
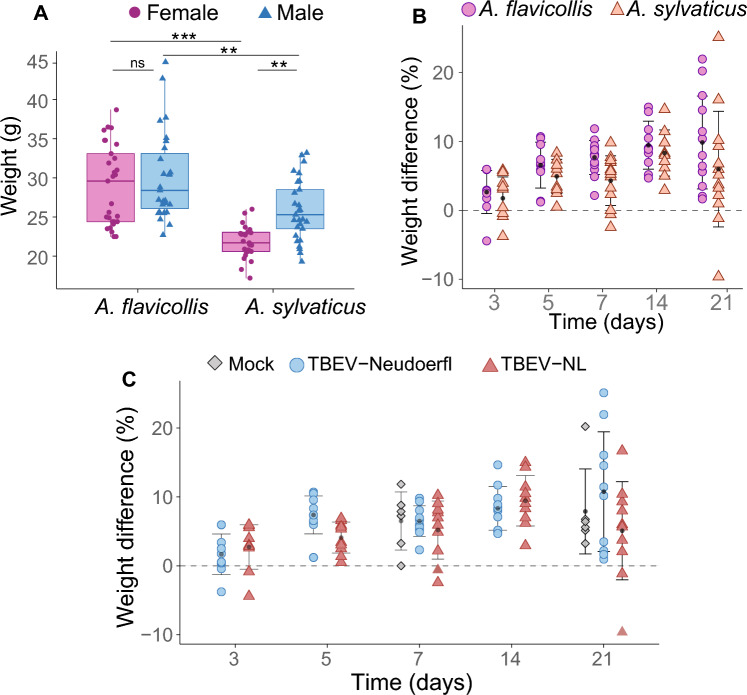
(**A**) Weight (g) of mice used for the study at the start of the infection experiment. *ns* not significant, **p < 0.01, ***p < 0.001. (**B**) Weight difference of animals relative to the start of the experiment per species from 3 to 21 days post inoculation (dpi). (**C**) Weight difference of *A. flavicollis* and *A. sylvaticus* relative to the start of the experiment of (mock) infected animals per treatment from 3 to 21 dpi.

Mice were weighed prior to inoculation with TBEV. Median weight for *A. flavicollis* males and females was 27.2 g and 30.2 g, respectively. Median weight for *A. sylvaticus* males and females was 24.9 g and 22.3 g, respectively. *Apodemus flavicollis* males and females were significantly heavier than *A. sylvaticus* males and females, respectively (Fig. 2A, Supplementary Table S1**)**. Furthermore, *A. sylvaticus* males were significantly heavier than female *A. sylvaticus* (Supplementary Table S1)*.*

Of importance, none of the *A. sylvaticus* and *A. flavicollis* mice showed signs of distress or clinical signs during the observation period. Furthermore, no apparent weight loss was observed in the majority of TBEV inoculated animals and only seven animals showed weight loss with a maximum loss of 10% during the experiment (Fig. 2B, C and Supplementary Table S2**)**. In fact, most mice gained weight between 3 and 21 dpi, most likely due to ad libitum feeding and controlled climate conditions. The animal with the 10% weight loss was infected with TBEV-NL, and only had detectable TBEV RNA in the blood and not in the organs. None of the mock-infected control mice experienced weight loss, as determined by measuring body weights at either 7 or 21 dpi.

### TBEV RNA detection in blood, spleen, liver and brain tissue

**Figure 3:**
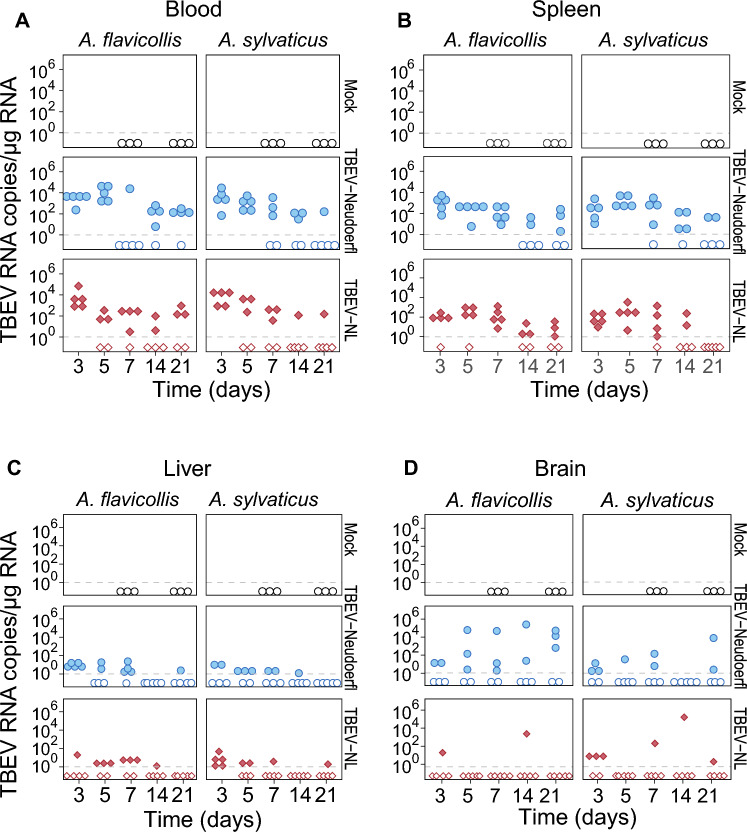
TBEV RNA copy numbers in mouse (**A**) whole blood, (**B**) spleen, (**C**) liver, (**D**) and brain tissue at different timepoints (days) after inoculation. RNA copy numbers were expressed as the number of TBEV RNA copy numbers per microgram (µg) of total RNA. The dashed lines indicate the limit of detection. Samples below this line are negative for TBEV RNA.

To assess TBEV infection in different mouse tissues following inoculation, we used an RT-PCR able to detect both TBEV strains in whole blood, spleen, liver and brain samples of the mice (Fig. 3A–D**, **Supplementary Table S3). TBEV RNA was detected in blood throughout the study period of 21 days and the presence or absence of TBEV RNA was not influenced by time after inoculation, TBEV strain, mouse species, the sex of the mice or the weight of the mice (GLMM, P > 0.05 for all variables, Fig. 3A, Supplementary Table S2). Nevertheless, we observed a decrease in TBEV RNA copy numbers from the start of inoculation until the end of the experiment (GLMM, P < 0.001).

TBEV RNA was detected in tissues throughout the study period, with viral RNA levels being lower in liver tissue compared to spleen tissue (Fig. 3B, C, Supplementary Table S2**)**. Furthermore, the proportion of mice with detectable TBEV RNA in the liver and spleen tissue decreased over time (GLMM, P < 0.05, Supplementary Table S2**)**. No effects of virus strain, mouse species or mouse weight on the likelihood of detecting TBEV RNA were found, however, TBEV RNA in the liver was more often found in females compared to males (GLMM, P < 0.05). In line with the blood samples, both the TBEV RNA copy numbers in liver and spleen samples decreased over time (GLMM, P < 0.05 and GLMM, P < 0.001, Fig. 3B,C and Supplementary Table S2**)**. No differences in TBEV RNA copy numbers between the TBEV strains were observed in liver tissue (Supplementary Table S2). In contrast, in spleen tissue, viral RNA copy numbers for TBEV-Neudoerfl were significantly higher compared to TBEV-NL (GLMM, P < 0.001) and were negatively related to the weight of the mice (GLMM, P < 0.01). There was no effect of mouse species on the TBEV RNA copy numbers.

The likelihood of TBEV RNA detection in the brain did not depend on the time after inoculation, or the sex, weight, or species of the mice. In contrast, the TBEV strain affected the presence of detectable TBEV RNA in mice brains. TBEV-Neudoerfl was more often detected in the brain compared to TBEV-NL (GLM, P < 0.01). Furthermore, TBEV RNA copy numbers in the brain increased over time (GLM, P < 0.01) and higher copy numbers were observed in the brains of *A. flavicollis* compared to *A. sylvaticus* (GLMM, P < 0.05, Fig. 3D) and in male mice compared to female mice (GLMM, P < 0.01, Supplementary Fig. S2**).** Overall, TBEV RNA could be detected in all tissue samples from 3 up to 21 dpi. Blood and spleen samples were the most likely to contain TBEV RNA compared to liver and brain tissue.

### Virus isolation from brain tissue and blood of mice

Virus isolation was attempted for 29 TBEV RNA-positive brain tissue samples. Interestingly, only male mice had high enough viral loads to successfully isolate the virus, roughly ≥ 5 × 10^3^ TBEV RNA copies/µg RNA and virus isolation attempts below this RNA level were unsuccessful. TBEV was successfully isolated from 7 out of 29 brain samples of mice inoculated with TBEV between 3 and 21 dpi (Supplementary Table S4). The majority of successful virus isolations (5) were from *A. flavicollis* males inoculated with TBEV-Neudoerfl. The other two successful isolations were from a male *A. flavicollis* and a male *A. sylvaticus*, both inoculated with TBEV-NL (Supplementary Table S4). We attempted virus isolation from 20 TBEV RNA-positive blood samples of mice euthanised between 3 and 21 days post infection but we were unsuccessful, due to either issues in sensitivity of the assay or potential interference of neutralizing antibodies (see next section).

### Antibody detection

**Figure 4:**
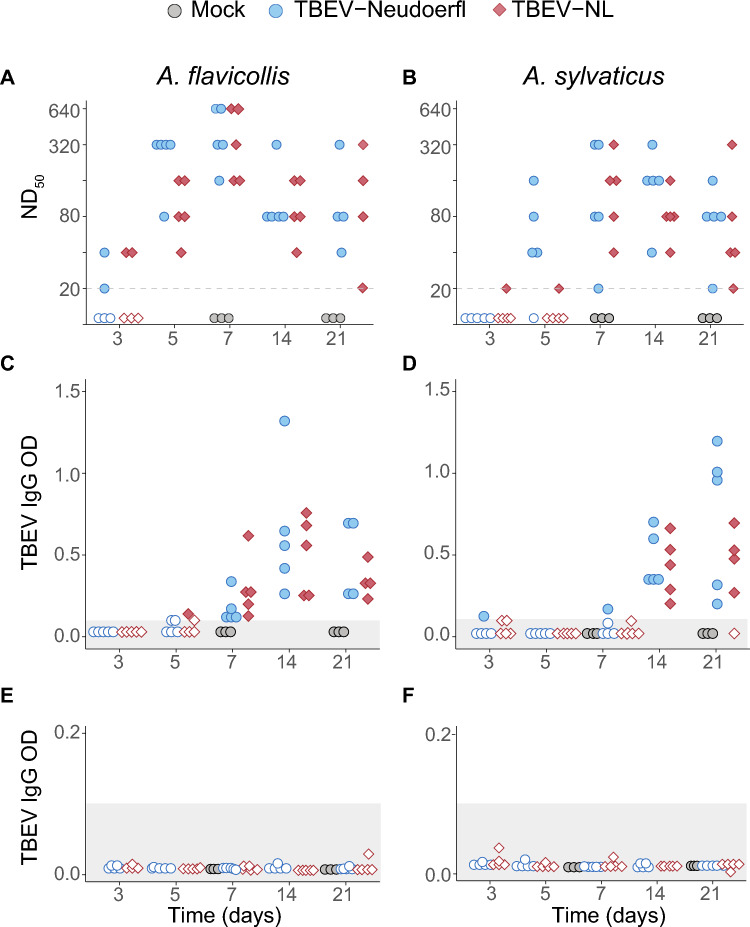
Neutralizing antibodies and TBEV IgG detection in TBEV infected *Apodemus* mice over time (days). A virus neutralization test was used to determine the neutralizing antibodies against TBEV in *A. flavicollis* (**A**) or *A. sylvaticus* (**B**). ND_50_: 50% neutralizing dose. The dashed line indicates the lower limit of detection of the neutralization assay at 20 ND_50_. IgG antibody levels against TBEV were determined using a TBEV IgG ELISA in (**C**) *A. flavicollis* or (**D**) *A. sylvaticus*. (**E,F**) The TBEV IgG ELISA result of mice before TBEV inoculation. An optical density (OD) of the mean plus three times the standard deviation of the negative controls was used as cut-off. The cut-off value is indicated by the shaded area. Dots represent individual mice for mock (grey), TBEV-Neudoerfl (blue) or TBEV-NL (red) inoculations for all panels.

We used virus neutralization tests (VNT) as well as an adapted commercial TBEV IgG ELISA to test for the presence of antibodies against TBEV. Neutralizing antibodies were detected as early as 3 dpi for both TBEV-NL and TBEV-Neudoerfl in both *Apodemus* species (Fig. 4A,B). The measured titers ranged from 20 to 640 ND_50_ between 3 and 21 dpi. All animals had neutralizing antibodies at 7 dpi except for one *A. sylvaticus* male infected with TBEV-Neudoerfl that was necropsied at 21 dpi. We were unable to collect serum from two *A. flavicollis* mice necropsied at 21 dpi because of insufficient blood draw. IgG ELISA suggested that all animals except one individual (male *A. sylvaticus* infected with TBEV-NL and necropsied at 21 dpi) had seroconverted after 14 dpi (Fig. 4C, D and Supplementary Table S4). The mock-inoculated mice were not positive for TBEV IgG nor did they have neutralizing antibodies against TBEV (Fig 4A-D, Supplementary Table S4**)**.

### Histopathology, immunohistochemistry and in situ hybridization of brain samples

**Figure 5:**
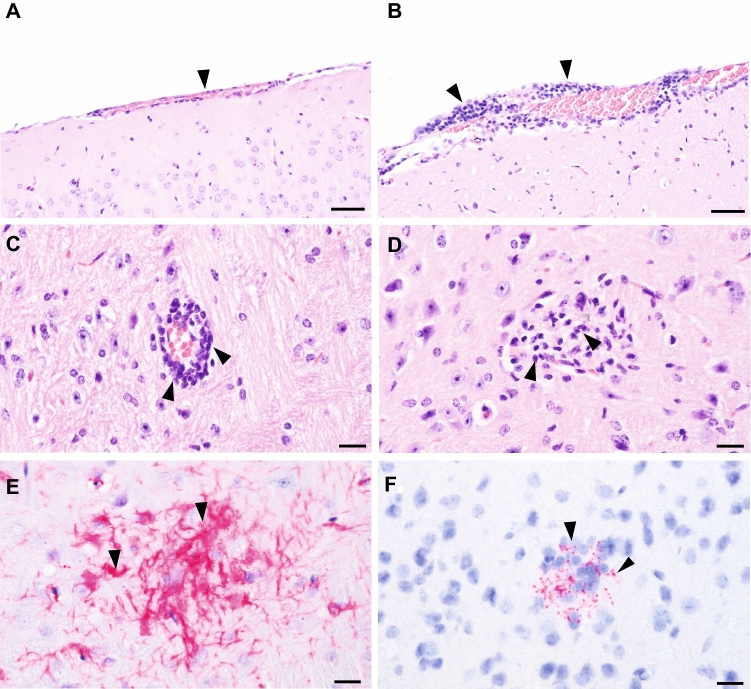
Histopathology of the brain of TBEV infected *Apodemus* mice. (**A**) Normal aspect of the meninges in a control mouse (arrow). (**B**) Meningitis in a TBEV-infected mouse showing increased number of mononuclear cells in the meninges (arrows). (**C**) Perivascular cuffing by mononuclear cells in the brain of TBEV-infected mouse (arrows). (**D,E**) Microglial nodule in TBEV-infected brain shown with hematoxylin–eosin (HE) staining (**D**, arrows) and anti-iba1 staining for microglia (**E**, arrows). (**F**)In situ hybridization showing the presence of TBEV RNA in TBEV infected mouse brain (arrows). Bar = 50 μm (**A,B**) or 20 μm (**C–F**).

We conducted histopathology, immunohistochemistry (IHC) and in situ hybridization (ISH) on selected mice with high levels of TBEV RNA copy numbers in the brain. All brain tissue of TBEV infected mice showed signs of a (meningo)-encephalitis, mononuclear perivascular cuffing and occasionally glial nodules (Fig. 5B–E). No viral proteins could be detected with IHC, but ISH showed localized granules of TBEV RNA in four out of 11 tested mice, sometimes coinciding with microglial nodules (Fig. 5F). However, in the four ISH positive mouse brains, the localized TBEV granules were only sporadically observed indicating low systemic viral replication in the brain. The mock infected mice did not show any histopathological alterations in the brain (Fig. 5A).

## Discussion

Rodents are important hosts in the transmission of TBEV, however, the relative contribution of different rodent species in the circulation and spread of different TBEV strains is not well understood. Here, we studied the infection dynamics of two TBEV strains in two wild mouse species, *A. sylvaticus* and *A. flavicollis*. The two species of mice were clinically unaffected after inoculation with either TBEV-Neudoerfl or TBEV-NL. Nevertheless, viral RNA was detected in whole blood and brain samples over a period of 21 days, showing the persistence of viral RNA in both mouse species.

TBEV RNA was detected throughout the study period of 21 days in all types of samples, i.e. blood, spleen, liver and brain tissue. Interestingly, an apparent peak in viraemia was not observed, in contrast to previous studies on *A.sylvaticus, A. flavicollis* and *M. glareolus*^19,23^. Most mice had TBEV neutralizing antibodies after 7 dpi and IgG antibodies against TBEV around 14 dpi, though some mice already presented with TBEV neutralizing antibodies from 3 dpi onwards. This early response is in line with a previous report in which *A. sylvaticus* had neutralizing antibodies against TBEV after 3 dpi^19^, but we cannot exclude these animals were primed in the field, despite that we did not detect IgG antibodies prior to TBEV inoculation. Previous work showed that TBEV RNA could be detected up to 28 days in whole blood samples of the bank vole *Myodes glareolus*^21^ and up to 120 days post TBEV inoculation in the northern red-backed vole *M. rutilus* and the striped wood mouse *A. agrarius*^24^*.* Furthermore, virus was successfully isolated from whole blood of *M. rutilus* after 60 dpi and from leukocytes of *M. glareolus* at 14 dpi^21,24^, both in the presence of neutralizing antibodies.

The presence of TBEV RNA in blood, liver and spleen tissue did not differ between mouse species and TBEV strains, however, differences in neuro-invasion were recorded. TBEV-Neudoerfl showed more efficient infection of the brain compared to TBEV-NL. Both viruses were detected for up to 21 days in the brain of inoculated mice. This is in contrast with a recent TBEV infection study with *Myodes glareolus* in which TBEV-Neudoerfl was not detected in the brain of voles after more than 7 days post inoculation, while other TBEV strains were detected in the brain of voles up to 28 days post inoculation using a similar inoculation dose and route^21,25^. Differences in the reservoir competence of hosts for different viral lineages have previously been shown for Powassan virus (POWV)^26^, a virus closely related to TBEV, thereby influencing the eco-epidemiology of the viral lineages.

Besides differences in the susceptibility of the host species, differences in neuroinvasive properties between TBEV-NL and TBEV-Neudoerfl could also be related to genetic variance of the virus strains, in particular in the 3’ untranslated regions (UTRs). The 3’UTR of flaviviruses, including TBEV, is known to influence virulence and neuroinvasion^27,28^. TBEV-NL has a truncated 3’UTR compared to TBEV-Neudoerfl^29^, which has a poly-A domain in the 3’UTR^30^. Insertion of poly-A in the 3’UTR of TBEV is known to increase virulence in mice^31^, possibly by increased evasion of viral sensing and type I interferon response^32^.

In addition to host and virus strain-specific effects, we also identified a sex-specific difference in susceptibility to infection. Male mice had significantly higher TBEV RNA loads in the brain compared to female mice. Furthermore, virus isolation from brain tissue was only successful for male mice. While we did not measure any immune parameters, the higher TBEV RNA loads in the males could reflect a difference in immune response to virus infection of male compared to female mice. Indeed, female mice often mount stronger immune responses and have a reduced susceptibility to virus infections compared to male mice^33,34^. Interestingly, male rodents may contribute more to the transmission of tick-borne pathogens than female rodents^35^, as a result of their higher activity and large home range^36^. As a consequence, male mice have higher tick burdens than females^37^. Furthermore, high testosterone levels are associated with decreased resistance to ticks^38^ and increased parasite loads in rodents^39^, thereby even further enhancing the contribution of male rodents to tick-borne pathogen transmission.

As both *Apodemus* species had similar TBEV infection rates in the blood for the two TBEV strains, this may indicate that *A. sylvaticus* and *A. flavicollis* are equally important for the transmission of TBEV. Nevertheless, host specific factors such as host activity and resistance to ticks could also influence their relative contribution to TBEV transmission since they influence the capacity of ticks to feed successfully on their host^3^. For example, yellow-necked mice have a higher tick burden compared to wood mice^40^, most likely the result of the higher body mass of the yellow-necked mice as observed in the current and other studies^40,41^. The higher tick burden of the yellow-necked mice could consequently result in a higher contribution to the transmission of TBEV either via viremic or non-viremic transmission.

The current work is a starting point to improve our understanding of the relative importance of viraemic versus non-viraemic transmission of TBEV from wild mice to ticks. In contrast to viraemic transmission, non-viraemic transmission is a recognized phenomenon experimentally tested in voles and mice^2^. However, the exact role of viraemic transmission of TBEV in natural hosts has gained little attention. The detection of persistent virus infections in leukocytes and the liver shows long-term presence of TBEV in experimentally infected rodents^7,21^. Furthermore, we detected viral RNA up to three weeks after infection in *Apodemus* mice. These studies may hint to the long-term presence of infectious TBEV in rodents. Nonetheless, the detected RNA levels in this study were low and may not reflect actual viraemia in the rodents as we were unsuccessful in isolating virus from a subset of TBEV RNA positive whole blood samples of mice. However, the level of viraemia needed for mouse-to-tick transmission is unknown and could even be below the detection limit of our assay. Therefore, transmission studies with ticks are needed to understand whether infectious virus still circulates in these rodents either as viraemia or cell-associated virus in leukocytes, and to test whether feeding ticks can become infected even after 2–4 dpi, as currently assumed^19,42,43^. During tick feeding, TBEV is recruited to the feeding site via migratory cells such as leukocytes^44^. This can result in the infection of ticks in the absence of detectable viremia in the blood, indicating that viremia is not a necessary condition for virus transmission^44^. Besides host-to-tick transmission, the absence of a natural tick-to-host transmission route could have influenced the infection success and virulence in the rodents. First, the inoculated dose of TBEV in the current study may not reflect the amount of virus particles transmitted by ticks. Second, the absence of tick saliva at the inoculation site may have altered the host response to virus infection as tick saliva promotes infection success and virulence in the vertebrate host^44–46^.

Laboratory mice are frequently used as model species in TBEV transmission experiments^47–49^, however, the biology of tick-borne viruses in their natural hosts is different compared to laboratory mice^50^. Laboratory mice show weight loss and hind limb paralysis after TBEV infection with often a lethal outcome^51,52^. In contrast, wild rodents do not show clear clinical signs, although sub-clinical encephalitis is observed^20,21^. In addition, we and others show meningoencephalitis in infected animals using immunohistochemical analyses^20,21^, as a result of TBEV infection in the brains. The differences in clinical responses between laboratory and wild mice are most likely caused by the activated state of the cellular immune system of wild rodents^53^. These differences observed between laboratory and wild mice underline the importance of using natural host species in viraemic and non-viraemic TBEV transmission studies.

## Conclusion

*Apodemus sylavticus* and *A. flavicollis* mice inoculated with either TBEV-NL or TBEV-Neudoerfl were TBEV RNA positive throughout the study period of 21 days, after which infectious virus could still be isolated from the brain. The results presented in the current study demonstrate that *A. flavicollis* and *A. sylvaticus* have a similar infection pattern to TBEV in the different tissues studied and that the presence of TBEV RNA in the blood after 21 days may indicate a longer viraemic period than previously shown. Future studies are however needed to determine the role of the induced neutralizing antibody response in the transmission potential of the virus and should assess if the low levels of TBEV RNA after 21 days reflects active viraemia. Finally, host-tick transmission experiments should be welcomed to assess the relative importance of viraemic and non-viraemic transmission in the eco-epidemiology of TBEV.

## Materials and methods

### Virus and cells

Human lung epithelial A549 cells were used for the preparation of virus stocks and end-point dilution assays (EPDA) and cultured in HEPES-buffered Dulbecco’s modified Eagle medium (DMEM; Gibco, Carlsbad, CA, USA) supplemented with 10% fetal bovine serum (FBS; Gibco) and 1% penicillin/streptomycin (Gibco) at 37 °C with 5% CO_2_. A549 cells used for virus isolation from brain samples (see below) were cultured as described but with the addition of Fungizone (2.5 μg/ml of amphotericin B and 2.1 μg/ml of sodium deoxycholate; Gibco) and Gentamycin (50 μg/ml; Gibco), subsequently named DMEM+. The absence of *Mycoplasma* infection in the A549 cell culture was verified using the MycoStrip™ detection kit (InvivoGen).

A P2 stock of TBEV-NL (GenBank accession no. ON502378.1) and a P4 stock of TBEV-Neudoerfl (GenBank accession no. U27495.1), kindly provided by the RIVM (Dutch National Health Institute), were used for subcutaneous inoculation of *A. sylvaticus* and *A. flavicollis*. Virus stocks were generated by infecting A549 cells at a multiplicity of infection of 0.01. Stocks were titrated on A549 cells in triplicate using end-point dilution assays according to Fros et al.^54^.

### Animal trapping and housing

Mice were trapped using Heslinga live traps (Heslinga, Groningen, the Netherlands). *Apodemus sylvaticus* were trapped in a forest plot near Wageningen and in two forest plots in the Twente region (Wageningen: 51°59′52.0″ N 5°42′43.4″ E, Twente region 52°27′04.2″ N 6°50′43.3″ E and 52°26′34.4″ N 6°52′57.6″ E), whereas *A. flavicollis* were trapped in the two forest plots in the Twente region. Traps were pre-baited for three consecutive nights with carrots, mealworms, grains and hay. Traps were armed after sunset and emptied 8–10 h later. *Apodemus* species were identified to species level based on the hind foot length together with the presence or absence of a yellow band on the neck of the animal. The hind foot length of *A. flavicollis* is generally larger than 22 mm whereas the hind foot length of *A. sylvaticus* ranges from 17-21 mm (^41^ and Supplementary Fig. S1**)**.

After transport from the field to the facilities at Wageningen University, animals were individually housed in large mesocosms before inoculation with TBEV. Mesocosms (70 × 90 × 40 cm, glass fiber) consisted of a layer (~ 5 to 10 cm) of sand and wood chips to mimic a natural situation together with hay and wooden branches (Fig. 1B). The mesocosms were closed with a 0.5 × 0.5 cm metal gauze lid and placed in a large farm shed exposed to the natural day-night light cycle between June and September. Mice were collected up to 1.5 months before the start of the experiment and housed for a maximum of four months in these mesocosms before transport to a BSL3 facility. They were individually transferred from the mesocosms into Makrolon type IIL cages three weeks before transport to the BSL3 facility. To rule out pre-exposure to TBEV, the mice were tested for TBEV IgG before inoculation as described below.

### TBEV infection in *Apodemus* species

Mice were individually housed in Makrolon type IIL cages in a BSL3 laboratory at 21 °C and ~ 50% RH. Animals were anaesthetised by placing a cage in a large induction box gassed with Sevoflurane (AbbVie, the Netherlands). Mice were weighed and subcutaneously inoculated with 10^5^ TCID_50_ in 100 µL of either TBEV-NL, TBEV-Neudoerfl or DMEM (mock inoculation).

Infected mice were monitored for up to 21 days, and necropsied at 3, 5, 7, 14 or 21 dpi. A total of 112 animals were used for the experiment; five mice per virus strain (TBEV-NL or TBEV-Neudoerfl) and per mouse species (*A. sylvaticus* or *A. flavicollis*) were necropsied at each of the five time points. Furthermore, three control animals per mouse species were necropsied at 7 and 21 dpi. Both male and female animals were used in the experiment and the fraction of males and females was similar for each virus strain or mouse species within one time point. This was either two males and three females or vice versa (Supplementary Table S4).

For blood and tissue sample collection at necropsy, animals were anaesthetised using Sevoflurane inhalation and ketamine/xylazine injected intra-peritoneally followed by exsanguination. Whole blood and serum were collected using MiniCollect EDTA tubes and MiniCollect serum tubes (Greiner, Austria), respectively. Tissue samples were collected from the animals and divided in two pieces (spleen and brain) or three pieces (liver) and frozen at −80 °C or fixed in 10% neutral buffered formalin for at least 48 h.

### Ethics statement

Animal experiments were conducted in accordance with European regulations (EU directive 2010/63/EU) and the Dutch Law on Animal Experiments (Wet op de dierproeven, ID number BWBR0003081). The animal experiments were approved by the Dutch Central Authority for Scientific Procedures on Animals (Permit Number: AVD1040020209209). All procedures were approved by the Animal Ethics Committees of Wageningen Research. The study was conducted in compliance with the ARRIVE guidelines for the reporting of animal studies^55^.

### RNA isolation

Total RNA from whole blood samples was isolated using the Mag-Bind Blood RNA kit (Omega Bio-tek) according to the manufacturer’s instructions: 200 µl whole blood was used per sample. Total RNA from mouse brain, spleen and liver samples was extracted using Trizol reagent (Invitrogen) according to the manufacturer’s instructions. Samples were first homogenized in DMEM+ with a Bullet Blender Storm (Next Advance) using 0.9–1.6 mm stainless steel beads at speed 10 for 2 min. For brain and liver samples, 500 µl of DMEM+ was added. For the spleen samples, 300 µl of DMEM+ was added. Samples were spun down for 1 min after homogenization and 100 µl homogenate was added to 1 ml Trizol. Total RNA yield was measured using a DS-11 spectrophotometer (DeNovix).

### RT-PCR

TBEV RNA was quantified using a real-time RT-PCR as described previously by Schwaiger and Cassinotti^56^. The reaction was performed in a CFX96 Real-Time PCR machine (Bio-Rad) with a 20 μl reaction system using the TaqMan RNA-to-CT 1 step Kit (Applied Biosystems).

An RNA standard to quantify TBEV RNA copy number was made based on a 83 bp amplicon of the 3’UTR of TBEV-Neudoerfl. cDNA was used as template to generate a specific PCR product using T7 primers (Supplementary Table S5) and the PCR product was verified by Sanger sequencing. The target sequence was transcribed in vitro using T7 polymerase with the T7 MEGAscript kit (Ambion) according to the manufacturer’s instruction. In vitro RNA was cleaned using conventional phenol/chloroform/isoamyl alcohol RNA extraction. RNA was quantified using a DS-11 spectrophotometer (DeNovix) in order to prepare a 10-time dilution series.

### Virus neutralization tests and TBEV IgG ELISA

A virus neutralization test (VNT) was performed on all serum samples using TBEV-Neudoerfl. Briefly, two-fold serial dilutions of sera (50 µl) were mixed with a fixed amount of virus (~ 100 TCID_50_ in 50 µl) in 96-well plates. After 2 h of incubation, 50,000 A549 cells (in 50 µl) were added to each well and plates were incubated for 5 days at 37 °C and 5% CO_2,_ and scored based on the presence of cytopathic effect (CPE). Neutralizing titres were determined using the Spearman–Kärber method^57,58^. Serum samples were considered positive at ND_50_ ≥ 1/20.

Sera from mice before inoculation as well as from TBEV-infected mice were incubated at 56 °C for 30 min in a heating block prior to serological analysis. EIA TBE Virus IgG (Testline, Brno, Czech Republic) was used according to the manufacturer’s instruction with the following modifications: mouse sera were diluted 1:50, and goat anti-mouse IgG antibody HRP conjugate (1:10,000) was used (Sigma-Aldrich, 2584AP181P). As a positive control, a serum found positive before was used. The cut-off value was calculated by adding three standard deviations (SD) to the mean optical density (OD) of the negative sera^59^.

### Virus isolation from brain and blood samples

Virus isolation was attempted from brain tissue using A549 cells. Briefly, A549 cells with a confluency of 70–80% were incubated with 100 µl brain homogenate or 50 µl of whole blood for 1.5 h. Cells were washed once with 1× PBS and checked for CPE after 4 days. When no CPE was visible, the cell supernatant was passaged for a maximum of three times.

### Histopathology and immunohistochemistry

Formalin-fixed tissues were routinely processed into paraffin blocks, cut into 4 µm sections, placed on positively charged glass slides (SuperfrostPlus®, Thermo Scientific) and dried for at least 48 h at 37 °C. After deparaffinization and rehydration in graded alcohols, sections were stained with hematoxylin–eosin (HE) or immunostained. For TBEV immunostaining, endogenous peroxidase was blocked for 30 min in methanol/H_2_O_2_, followed by antigen retrieval with 0.1% trypsin in Tris-buffered saline for 30 min at 37 °C. The monoclonal antibody 12292 (Native Antigen Company, Oxford, UK) directed against the NS1 protein of TBEV was used as the primary antibody at a dilution of 1:100 and incubated for 1 h. HRP conjugated anti-mouse IgG polymer (Invitrogen, Carlsbad, USA) was used as secondary antibody and incubated for 30 min followed by incubation with DAB + substrate (Agilent, Santa Clara, USA) for 5 min.

For immunostaining of microglia, sections were pre-treated by autoclaving at 121 °C in citrate buffer of pH 6 (Antigen unmasking solution, Vector Laboratories, Peterborough, UK)) for 5 min. The primary antibody used was the rabbit polyclonal anti-IBA-1 (Fujifilm Cellular Dynamics, Madison, WI, USA) diluted 1:500 and incubated for 1 h. Alkaline phosphatase conjugated anti-rabbit IgG polymer (Vector laboratories) was used as secondary antibody and incubated for 30 min followed by incubation with ImmPACT VectorRed substrate (Vector Laboratories) for 20 min at 37 °C. Sections were briefly counterstained with hematoxylin, dehydrated, and mounted permanently.

### In situ hybridization

For detection of TBEV RNA by RNAscope^®^ (Bio-Techne Ltd. Abingdon, UK) sections were deparaffinized in xylene and rehydrated in graded alcohols. After pre-treatment by boiling in RNAscope^®^ target retrieval reagent for 15 min followed by 30 min incubation with RNAscope^®^ Protease plus at 40 °C, sections were hybridized with the RNAscope^®^ probe pair V-TBEV-NS3. The probes were further hybridized to a cascade of signal amplification molecules according to the manufacturer’s instructions and signal was developed using the Fast Red substrate. Sections were briefly counterstained with hematoxylin, dried and mounted permanently.

### Statistical analyses

A linear model (LM) with a log-link function was used to test the associations of sex and mouse species with the weight of the mouse, including an interaction term between mouse and sex. A generalized linear model (GLM) with a log-link function was used to test for an effect of sex and mouse species on hindfoot length of the mouse. GLMs with a binomial distribution and logit-link function were used to evaluate the effects of virus strain, mouse species, time after infection, mouse sex and mouse weight on the presence/absence of TBEV RNA. GLMs with a negative binomial distribution and log-link function were used to test the effect of virus strain, mouse species, time after infection, mouse sex and mouse weight on TBEV RNA copy numbers. Model diagnostics were performed using the *DHARMa* package^60^. (G)LMs were constructed using the *glmmTMB* package^61^. Estimated marginal mean infection rates and TBEV RNA copy numbers were calculated using the *emmeans* package^62^. Pairwise contrasts of significant effects were performed with a Tukey HSD. All statistical analyses were carried out with the statistical software package R version 4.2.0 using Rstudio^63^.

### Supplementary Information

Supplementary Information.

## Data Availability

All data generated or analysed during this study are included in this published article (and its supplementary information files).
