# Supplementary material for: Infection of wild-caught wood mice (Apodemus sylvaticus) and yellow-necked mice (A. flavicollis) with tick-borne encephalitis virus
Source: Sci Rep. 2023 Dec 7;13:21627. doi: 10.1038/s41598-023-47697-2 (PMC10703896; doi:10.1038/s41598-023-47697-2)
Supplement: Supplementary file 1 — Supplementary Information. [file 41598_2023_47697_MOESM1_ESM.pdf]

Supplementary data

Infection of wild-caught wood mice (*Apodemus sylvaticus*) and yellow-necked mice (*A. flavicollis*) with tick-borne encephalitis virus

Julian W Bakker[1], Emily L Pascoe[1,2], Sandra van de Water[3], Lucien van Keulen[4], Ankje de Vries[5], Lianne C Woudstra[6], Helen J Esser[6], Gorben P Pijlman[7], Willem F de Boer[6], Hein Sprong[5], Jeroen Kortekaas[3,7,8] , Paul J Wichgers Schreur[3], Constantianus JM Koenraadt[1]

[1]Laboratory of Entomology, Wageningen University & Research, Wageningen, the Netherlands

[2] Conservation Genomics Research Unit, Research and Innovation Centre, Fondazione Edmund Mach, Trento, Italy

[3]Department of Virology and Molecular Biology,, Wageningen Bioveterinary Research, Lelystad, The Netherlands

[4]Department of Bacteriology, Host-Pathogen Interaction and Diagnostics Development, Wageningen Bioveterinary Research, Lelystad, The Netherlands

[5]National Institute of Public Health and the Environment (RIVM), Utrecht, the Netherlands

[6]Wildlife Ecology and Conservation Group, Wageningen University & Research, Wageningen, the Netherlands

[7]Laboratory of Virology, Wageningen University & Research, Wageningen, the Netherlands

[8]Present address: Boehringer Ingelheim Animal Health, Saint Priest, France

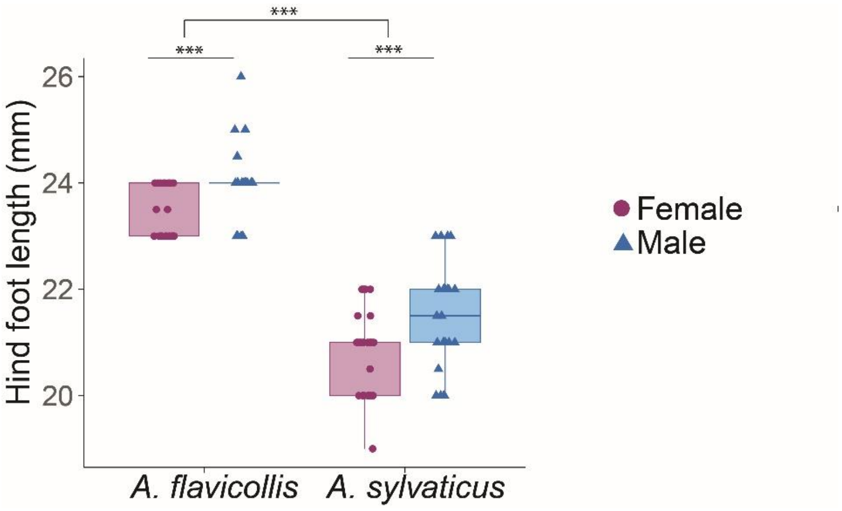

Figure S1. Hindfoot length (mm) of *Apodemus flavicollis* and *Apodemus sylvaticus* used in this study. The length of the hindfoot of male mice was significantly longer compared to female mice for both species (GLMM, LRT, χ^2 = 19.5, p < 0.001) and significantly longer for *A. flavicollis* males and females than for *A. sylvaticus* males and females, respectively (GLMM, LRT, χ^2 = 157.9, p < 0.001).

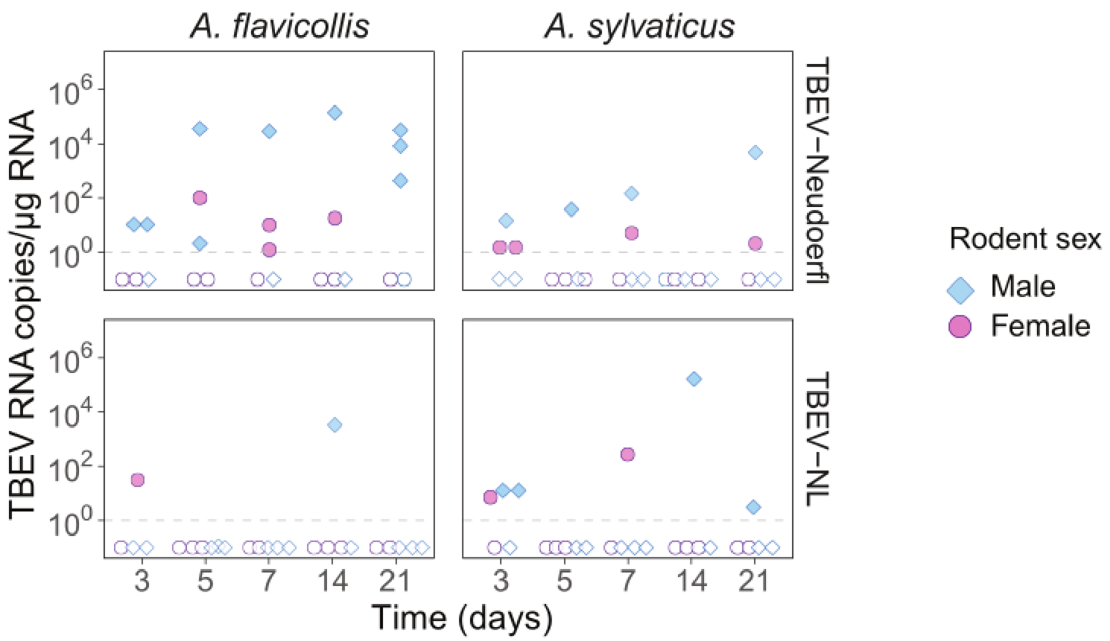

Figure S2. TBEV RNA copy numbers in mouse brain tissue at different timepoints (days) after inoculation by rodent sex. RNA copy numbers were expressed as the number of TBEV RNA copy numbers per microgram (µg) of total RNA. The dashed lines indicate the limit of detection. Samples below this line are negative for TBEV RNA.

Table S1. Incidence rate ratio (IRR) of weight of *A. flavicollis* and *A. sylvaticus*

**Species (Male [M] or Female [F])**		**IRR**	**95% CI**	**P**
A. flavicollis M	*A. flavicollis* F	0.963	0.84-1.07	0.77
A. flavicollis M	*A. sylvaticus* M	1.16	1.05-1.29	< 0.01
A. flavicollis F	*A. sylvaticus* F	1.32	1.17-1.50	< 0.001
A. sylvaticus F	*A. sylvaticus* M	0.85	0.74-0.96	< 0.01

Table S2. Likelihood ration test results of Generalized Linear Mixed Models for TBEV RNA presence/absence and copy numbers of TBEV RNA.

**Variable**	**Model**	**df**	χ^2	**P**
**TBEV RNA detection (**Binomial ~ logit-link)				
Blood	Timepoint	4	6.25	0.1
	Sex	1	3.16	0.07
	Weight	1	3.78	0.05
	Virus	1	2.29	0.13
Spleen	Timepoint	4	29.14	<0.001
Liver	Timepoint	4	26.15	<0.001
	Sex	1	4.29	<0.05
Brain	Virus	1	8.8	<0.01
	Sex	1	2.29	0.12
**TBEV RNA copy number** (Negative binomial ~ log-link)				
Blood RNA copy number	days post infection	4	45.17	< 0.001
	Sex	1	2.66	0.1
	Weight	1	5.99	<0.05
Liver RNA copy number	days post infection	4	12.09	<0.05
	Sex	1	3.17	0.07
	Species	1	3.31	0.07
Spleen RNA copy number	days post infection	4	27.41	<0.001
	Virus	1	15.48	<0.001
	Weight	1	7.56	<0.01
Brain RNA copy number	days post infection	4	16.21	<0.01
	Virus	1	2.39	0.12
	Species	1	3.99	<0.05
	Sex	1	7.12	<0.01

Table S3. Detection rates of TBEV RNA in blood, spleen, liver and brain samples

	TBEV NL		TBEV-Neudoerfl	
**Blood**	***A. flavicollis***	***A. sylvaticus***	***A. flavicollis***	***A. sylvaticus***
**3**	5/5	5/5	5/5	5/5
**5**	3/5	3/5	5/5	5/5
**7**	4/5	3/5	1/5	3/5
**14**	2/5	1/5	4/5	3/5
**21**	3/5	1/5	4/5	1/5
**Spleen**				
**3**	4/5	5/5	5/5	5/5
**5**	4/5	5/5	5/5	5/5
**7**	5/5	4/5	5/5	4/5
**14**	3/5	2/5	2/5	4/5
**21**	3/5	-/5	3/5	2/5
**Liver**				
**3**	1/5	5/5	5/5	2/5
**5**	3/5	2/5	2/5	3/5
**7**	3/5	1/5	4/5	2/5
**14**	1/5	-/5	-/5	1/5
**21**	-/5	1/5	1/5	-/5
**Brain**				
**3**	1/5	3/5	2/5	3/5
**5**	-/5	-/5	3/5	1/5
**7**	-/5	1/5	3/5	2/5
**14**	1/5	1/5	2/5	-/5
**21**	-/5	1/5	3/5	2/5

– indicates absence of TBEV RNA.

Table S4. Overview of *Apodemus* mice used in the experiment.

Mouse_ID	Virus	Timepoint	*Species*	Sex	VNT ND_{50}	IgG TBEV before TBEV infection	IgG TBEV after TBEV infection	TBEV RNA in blood	TBEV RNA spleen	TBEV RNA Liver	TBEV RNA brain*	Weight start experiment	Weight end experiment	Weight difference
TBEV 1.1	TBEV-NL	3	*A. sylvaticus*	Female	0	Neg	Neg	Pos	Pos	Pos	Pos	22.7	22.5	-1%
TBEV 1.2	TBEV-NL	3	*A. sylvaticus*	Male	0	Neg	Neg	Pos	Pos	Pos	Neg	25.9	26.7	3%
TBEV 1.3	TBEV-NL	3	*A. sylvaticus*	Female	20	Neg	Neg	Pos	Pos	Pos	Neg	17.2	18.2	6%
TBEV 1.4	TBEV-NL	3	*A. sylvaticus*	Male	0	Neg	Neg	Pos	Pos	Pos	Pos	25.3	26.3	4%
TBEV 1.5	TBEV-NL	3	*A. sylvaticus*	Male	0	Neg	Neg	Pos	Pos	Pos	Pos	23.5	24.8	6%
TBEV 1.6	TBEV-NL	5	*A. sylvaticus*	Male	0	Neg	Neg	Pos	Pos	Neg	Neg	24.1	24.7	2%
TBEV 1.7	TBEV-NL	5	*A. sylvaticus*	Female	0	Neg	Neg	Pos	Pos	Pos	Neg	20.9	21.0	0%
TBEV 1.8	TBEV-NL	5	*A. sylvaticus*	Female	0	Neg	Neg	Neg	Pos	Neg	Neg	20.6	21.3	3%
TBEV 1.9	TBEV-NL	5	*A. sylvaticus*	Female	20	Neg	Neg	Neg	Pos	Neg	Neg	23.1	23.8	3%
TBEV 1.10	TBEV-NL	5	*A. sylvaticus*	Male	0	Neg	Neg	Pos	Pos	Pos	Neg	28.1	29.6	5%
TBEV 1.11	TBEV-NL	7	*A. sylvaticus*	Female	80	Neg	Neg	Pos	Neg	Neg	Pos	23.4	25.2	8%
TBEV 1.12	TBEV-NL	7	*A. sylvaticus*	Male	40	Neg	Neg	Pos	Pos	Neg	Neg	24.6	25.8	5%
TBEV 1.13	TBEV-NL	7	*A. sylvaticus*	Female	160	Neg	Neg	Neg	Pos	Pos	Neg	23.0	24.6	7%
TBEV 1.14	TBEV-NL	7	*A. sylvaticus*	Male	320	Neg	Neg	Pos	Pos	Neg	Neg	32.9	32.7	-1%
TBEV 1.15	TBEV-NL	7	*A. sylvaticus*	Male	160	Neg	Neg	Neg	Pos	Neg	Neg	24.4	23.8	-2%
TBEV 1.16	TBEV-NL	14	*A. sylvaticus*	Female	40	Neg	Pos	Neg	Neg	Neg	Neg	24.3	25.8	6%
TBEV 1.17	TBEV-NL	14	*A. sylvaticus*	Female	80	Neg	Pos	Pos	Neg	Neg	Neg	20.6	21.2	3%
TBEV 1.18	TBEV-NL	14	*A. sylvaticus*	Male	80	Neg	Pos	Neg	Pos	Neg	Neg	20.4	22.4	10%
TBEV 1.19	TBEV-NL	14	*A. sylvaticus*	Male	80	Neg	Pos	Neg	Neg	Neg	Pos	30.6	34.1	11%
TBEV 1.20	TBEV-NL	14	*A. sylvaticus*	Female	160	Neg	Pos	Neg	Pos	Neg	Neg	21.7	23.5	8%
TBEV 1.21	TBEV-NL	21	*A. sylvaticus*	Female	320	Neg	Pos	Neg	Neg	Neg	Neg	20.1	21.3	6%
TBEV 1.22	TBEV-NL	21	*A. sylvaticus*	Female	80	Neg	Pos	Neg	Neg	Pos	Neg	21.5	22.3	4%
TBEV 1.23	TBEV-NL	21	*A. sylvaticus*	Male	40	Neg	Pos	Neg	Neg	Neg	Neg	22.6	24.7	9%
TBEV 1.24	TBEV-NL	21	*A. sylvaticus*	Male	20	Neg	Pos	Pos	Neg	Neg	Neg	33.2	30.0	-10%
TBEV 1.25	TBEV-NL	21	*A. sylvaticus*	Male	40	Neg	Neg	Neg	Neg	Neg	Pos	25.8	25.5	-1%
TBEV 2.1	TBEV-Neudoerfl	3	*A. sylvaticus*	Female	0	Neg	Neg	Pos	Pos	Neg	Pos	26.0	26.1	0%
TBEV 2.2	TBEV-Neudoerfl	3	*A. sylvaticus*	Male	0	Neg	Neg	Pos	Pos	Neg	Neg	21.2	20.4	-4%
TBEV 2.3	TBEV-Neudoerfl	3	*A. sylvaticus*	Female	0	Neg	Neg	Pos	Pos	Pos	Pos	23.7	23.6	0%
TBEV 2.4	TBEV-Neudoerfl	3	*A. sylvaticus*	Male	0	Neg	Neg	Pos	Pos	Neg	Neg	29.5	30.5	3%
TBEV 2.5	TBEV-Neudoerfl	3	*A. sylvaticus*	Male	0	Neg	Pos	Pos	Pos	Pos	Pos	24.6	24.7	0%
TBEV 2.6	TBEV-Neudoerfl	5	*A. sylvaticus*	Male	40	Neg	Neg	Pos	Pos	Pos	Pos	22.0	23.4	6%
TBEV 2.7	TBEV-Neudoerfl	5	*A. sylvaticus*	Female	160	Neg	Neg	Pos	Pos	Pos	Neg	22.8	24.7	8%
TBEV 2.8	TBEV-Neudoerfl	5	*A. sylvaticus*	Female	80	Neg	Neg	Pos	Pos	Pos	Neg	20.5	22.0	7%
TBEV 2.9	TBEV-Neudoerfl	5	*A. sylvaticus*	Male	0	Neg	Neg	Pos	Pos	Neg	Neg	26.7	28.3	6%
TBEV 2.10	TBEV-Neudoerfl	5	*A. sylvaticus*	Female	40	Neg	Neg	Pos	Pos	Neg	Neg	18.3	19.5	7%
TBEV 2.11	TBEV-Neudoerfl	7	*A. sylvaticus*	Female	320	Neg	Neg	Pos	Pos	Pos	Neg	21.9	23.2	6%
TBEV 2.12	TBEV-Neudoerfl	7	*A. sylvaticus*	Male	80	Neg	Neg	Pos	Pos	Neg	Pos	20.9	22.0	5%
TBEV 2.13	TBEV-Neudoerfl	7	*A. sylvaticus*	Female	320	Neg	Neg	Neg	Pos	Neg	Pos	22.9	24.2	6%
TBEV 2.14	TBEV-Neudoerfl	7	*A. sylvaticus*	Male	80	Neg	Pos	Neg	Neg	Pos	Neg	25.9	26.5	2%
TBEV 2.15	TBEV-Neudoerfl	7	*A. sylvaticus*	Male	20	Neg	Neg	Pos	Pos	Neg	Neg	26.5	29.1	10%
TBEV 2.16	TBEV-Neudoerfl	14	*A. sylvaticus*	Female	320	Neg	Pos	Pos	Pos	Neg	Neg	25.5	28.0	10%
TBEV 2.17	TBEV-Neudoerfl	14	*A. sylvaticus*	Male	160	Neg	Pos	Neg	Pos	Neg	Neg	23.7	25.6	8%
TBEV 2.18	TBEV-Neudoerfl	14	*A. sylvaticus*	Male	160	Neg	Pos	Neg	Pos	Neg	Neg	32.1	33.7	5%
TBEV 2.19	TBEV-Neudoerfl	14	*A. sylvaticus*	Female	40	Neg	Pos	Pos	Neg	Pos	Neg	19.7	21.1	7%
TBEV 2.20	TBEV-Neudoerfl	14	*A. sylvaticus*	Female	160	Neg	Pos	Pos	Pos	Neg	Neg	23.2	26.6	15%
TBEV 2.21	TBEV-Neudoerfl	21	*A. sylvaticus*	Female	80	Neg	Pos	Neg	Pos	Neg	Neg	20.8	21.0	1%
TBEV 2.22	TBEV-Neudoerfl	21	*A. sylvaticus*	Female	80	Neg	Pos	Pos	Neg	Neg	Pos	22.8	23.3	2%
TBEV 2.23	TBEV-Neudoerfl	21	*A. sylvaticus*	Male	160	Neg	Pos	Neg	Neg	Neg	Pos	21.9	27.4	25%
TBEV 2.24	TBEV-Neudoerfl	21	*A. sylvaticus*	Male	20	Neg	Pos	Neg	Neg	Neg	Neg	28.5	31.4	10%
TBEV 2.25	TBEV-Neudoerfl	21	*A. sylvaticus*	Male	80	Neg	Pos	Neg	Pos	Neg	Neg	19.3	22.4	16%
TBEV 3.1	Mock	7	*A. sylvaticus*	Female	0	Neg	Neg	Neg	Neg	Neg	Neg	21.5	22.2	3%
TBEV 3.2	Mock	7	*A. sylvaticus*	Female	0	Neg	Neg	Neg	Neg	Neg	Neg	19.3	20.7	7%
TBEV 3.3	Mock	7	*A. sylvaticus*	Male	0	Neg	Neg	Neg	Neg	Neg	Neg	30.9	30.9	0%
TBEV 3.4	Mock	21	*A. sylvaticus*	Female	0	Neg	Neg	Neg	Neg	Neg	Neg	21.4	22.5	5%
TBEV 3.5	Mock	21	*A. sylvaticus*	Male	0	Neg	Neg	Neg	Neg	Neg	Neg	24.7	25.5	3%
TBEV 3.6	Mock	21	*A. sylvaticus*	Male	0	Neg	Neg	Neg	Neg	Neg	Neg	29.6	31.6	7%
TBEV 4.1	TBEV-NL	3	*A. flavicollis*	Male	40	Neg	Neg	Pos	Pos	Neg	Neg	33.8	32.3	-4%
TBEV 4.2	TBEV-NL	3	*A. flavicollis*	Male	40	Neg	Neg	Pos	Pos	Neg	Neg	30.7	31.6	3%
TBEV 4.3	TBEV-NL	3	*A. flavicollis*	Female	0	Neg	Neg	Pos	Neg	Pos	Pos	38.7	39.7	3%
TBEV 4.4	TBEV-NL	3	*A. flavicollis*	Female	0	Neg	Neg	Pos	Pos	Neg	Neg	25.0	25.7	3%
TBEV 4.5	TBEV-NL	3	*A. flavicollis*	Male	0	Neg	Neg	Pos	Pos	Neg	Neg	27.0	28.6	6%
TBEV 4.6	TBEV-NL	5	*A. flavicollis*	Male	160	Neg	Neg	Neg	Pos	Pos	Neg	35.1	37.4	7%
TBEV 4.7	TBEV-NL	5	*A. flavicollis*	Male	80	Neg	Neg	Neg	Neg	Neg	Neg	25.5	27.0	6%
TBEV 4.8	TBEV-NL	5	*A. flavicollis*	Female	40	Neg	Pos	Pos	Pos	Neg	Neg	36.4	38.9	7%
TBEV 4.9	TBEV-NL	5	*A. flavicollis*	Female	160	Neg	Neg	Pos	Pos	Pos	Neg	31.9	33.7	6%
TBEV 4.10	TBEV-NL	5	*A. flavicollis*	Female	80	Neg	Neg	Pos	Pos	Pos	Neg	22.5	22.8	1%
TBEV 4.11	TBEV-NL	7	*A. flavicollis*	Male	640	Neg	Pos	Pos	Pos	Neg	Neg	30.5	32.3	6%
TBEV 4.12	TBEV-NL	7	*A. flavicollis*	Male	320	Neg	Pos	Neg	Pos	Neg	Neg	27.2	29.4	8%
TBEV 4.13	TBEV-NL	7	*A. flavicollis*	Female	160	Neg	Pos	Pos	Pos	Pos	Neg	32.5	33.2	2%

TBEV 4.14	TBEV-NL	7	*A. flavicollis*	Female	160	Neg	Pos	Pos	Pos	Pos	Neg	22.5	24.8	10%
TBEV 4.15	TBEV-NL	7	*A. flavicollis*	Male	640	Neg	Pos	Pos	Pos	Pos	Neg	26.6	29.0	9%
TBEV 4.16	TBEV-NL	14	*A. flavicollis*	Male	160	Neg	Pos	Neg	Pos	Neg	Neg	24.7	28.4	15%
TBEV 4.17	TBEV-NL	14	*A. flavicollis*	Male	160	Neg	Pos	Pos	Pos	Pos	Pos	37.7	43.1	14%
TBEV 4.18	TBEV-NL	14	*A. flavicollis*	Female	40	Neg	Pos	Neg	Neg	Neg	Neg	31.0	33.8	9%
TBEV 4.19	TBEV-NL	14	*A. flavicollis*	Female	80	Neg	Pos	Neg	Pos	Neg	Neg	24.4	26.1	7%
TBEV 4.20	TBEV-NL	14	*A. flavicollis*	Female	80	Neg	Pos	Pos	Neg	Neg	Neg	29.6	32.7	10%
TBEV 4.21	TBEV-NL	21	*A. flavicollis*	Male	80	Neg	Pos	Neg	Neg	Neg	Neg	32.4	37.8	17%
TBEV 4.22	TBEV-NL	21	*A. flavicollis*	Female	20	Neg	Pos	Pos	Neg	Neg	Neg	34.3	35.0	2%
TBEV 4.23	TBEV-NL	21	*A. flavicollis*	Male	160	Neg	Pos	Pos	Pos	Neg	Neg	26.6	28.1	6%
TBEV 4.24	TBEV-NL	21	*A. flavicollis*	Female	na	Neg	na	Pos	Pos	Neg	Neg	23.1	25.5	10%
TBEV 4.25	TBEV-NL	21	*A. flavicollis*	Male	320	Neg	Pos	Neg	Pos	Neg	Neg	42.7	46.1	8%
TBEV 5.1	TBEV-Neudoerfl	3	*A. flavicollis*	Male	20	Neg	Neg	Pos	Pos	Pos	Neg	30.5	32.3	6%
TBEV 5.2	TBEV-Neudoerfl	3	*A. flavicollis*	Male	0	Neg	Neg	Pos	Pos	Pos	Pos	22.7	23.1	2%
TBEV 5.3	TBEV-Neudoerfl	3	*A. flavicollis*	Female	40	Neg	Neg	Pos	Pos	Pos	Neg	36.5	36.7	1%
TBEV 5.4	TBEV-Neudoerfl	3	*A. flavicollis*	Female	0	Neg	Neg	Pos	Pos	Pos	Neg	23.5	24.9	6%
TBEV 5.5	TBEV-Neudoerfl	3	*A. flavicollis*	Male	0	Neg	Neg	Pos	Pos	Pos	Pos	24.0	24.6	3%
TBEV 5.6	TBEV-Neudoerfl	5	*A. flavicollis*	Male	80	Neg	Neg	Pos	Pos	Neg	Pos	27.1	30.0	11%
TBEV 5.7	TBEV-Neudoerfl	5	*A. flavicollis*	Male	320	Neg	Neg	Pos	Pos	Neg	Pos	44.8	49.5	10%
TBEV 5.8	TBEV-Neudoerfl	5	*A. flavicollis*	Female	320	Neg	Neg	Pos	Pos	Pos	Neg	24.4	26.2	7%
TBEV 5.9	TBEV-Neudoerfl	5	*A. flavicollis*	Female	320	Neg	Neg	Pos	Pos	Pos	Pos	25.1	27.5	10%
TBEV 5.10	TBEV-Neudoerfl	5	*A. flavicollis*	Female	320	Neg	Neg	Pos	Pos	Neg	Neg	33.1	33.5	1%
TBEV 5.11	TBEV-Neudoerfl	7	*A. flavicollis*	Female	320	Neg	Pos	Pos	Pos	Pos	Pos	34.8	37.6	8%
TBEV 5.12	TBEV-Neudoerfl	7	*A. flavicollis*	Male	640	Neg	Pos	Neg	Pos	Pos	Neg	34.7	37.0	7%
TBEV 5.13	TBEV-Neudoerfl	7	*A. flavicollis*	Female	160	Neg	Neg	Neg	Pos	Neg	Pos	30.7	32.2	5%
TBEV 5.14	TBEV-Neudoerfl	7	*A. flavicollis*	Female	320	Neg	Pos	Neg	Pos	Pos	Neg	24.4	26.1	7%
TBEV 5.15	TBEV-Neudoerfl	7	*A. flavicollis*	Male	640	Neg	Pos	Neg	Pos	Pos	Pos	25.6	28.0	9%
TBEV 5.16	TBEV-Neudoerfl	14	*A. flavicollis*	Male	80	Neg	Pos	Pos	Pos	Neg	Pos	28.8	31.2	8%
TBEV 5.17	TBEV-Neudoerfl	14	*A. flavicollis*	Female	80	Neg	Pos	Pos	Pos	Neg	Neg	34.8	36.6	5%
TBEV 5.18	TBEV-Neudoerfl	14	*A. flavicollis*	Male	80	Neg	Pos	Neg	Neg	Neg	Neg	25.6	28.6	12%
TBEV 5.19	TBEV-Neudoerfl	14	*A. flavicollis*	Female	80	Neg	Pos	Pos	Neg	Neg	Neg	36.1	39.3	9%
TBEV 5.20	TBEV-Neudoerfl	14	*A. flavicollis*	Female	320	Neg	Pos	Pos	Neg	Neg	Pos	25.6	26.8	5%
TBEV 5.21	TBEV-Neudoerfl	21	*A. flavicollis*	Male	320	Neg	Pos	Pos	Pos	Neg	Pos	37.3	38.6	3%
TBEV 5.22	TBEV-Neudoerfl	21	*A. flavicollis*	Female	80	Neg	Pos	Pos	Pos	Neg	Neg	30.2	30.7	2%
TBEV 5.23	TBEV-Neudoerfl	21	*A. flavicollis*	Female	40	Neg	Pos	Neg	Pos	Pos	Neg	23.6	26.3	11%
TBEV 5.24	TBEV-Neudoerfl	21	*A. flavicollis*	Male	80	Neg	Pos	Pos	Neg	Neg	Pos	26.8	30.7	15%
TBEV 5.25	TBEV-Neudoerfl	21	*A. flavicollis*	Male	na	Neg	na	Pos	Neg	Neg	Pos	25.5	31.1	22%
TBEV 6.1	Mock	7	*A. flavicollis*	Female	0	Neg	Neg	Neg	Neg	Neg	Neg	24.1	26.0	8%
TBEV 6.2	Mock	7	*A. flavicollis*	Female	0	Neg	Neg	Neg	Neg	Neg	Neg	30.4	34.0	12%
TBEV 6.3	Mock	7	*A. flavicollis*	Male	0	Neg	Neg	Neg	Neg	Neg	Neg	28.4	30.9	9%
TBEV 6.4	Mock	21	*A. flavicollis*	Male	0	Neg	Neg	Neg	Neg	Neg	Neg	30.4	32.1	6%
TBEV 6.5	Mock	21	*A. flavicollis*	Female	0	Neg	Neg	Neg	Neg	Neg	Neg	24.9	26.5	6%
TBEV 6.6	Mock	21	*A. flavicollis*	Female	0	Neg	Neg	Neg	Neg	Neg	Neg	26.7	32.1	20%

*Green boxes of TBEV RNA positive brain samples indicate successful virus isolation on A549 cells. "na" indicates insufficient samples for analysis.

Table S5.T7 primers for in vitro RNA synthesis of the 3'UTR of TBEV

Primer	Sequence
TBEV_3'UTR_T7	TAATACGACTCACTATAGGGAGGGGGGCGGTTCTTG
TBEV_3'UTR_R	TTTCCGAGTCACACATCACCTC
TBEV_3'UTR_F	AGGGGGGGCGGTTCTTG
